# Levels of Selected Matrix Metalloproteinases—MMP-1, MMP-2 and Fibronectin in the Saliva of Patients Planned for Endodontic Treatment or Surgical Extraction

**DOI:** 10.3390/jcm9123971

**Published:** 2020-12-07

**Authors:** Ewa Matuszczak, Izabela Cwalina, Marzena Tylicka, Katarzyna Wawrzyn, Magdalena Nowosielska, Anna Sankiewicz, Łukasz Ołdak, Ewa Gorodkiewicz, Adam Hermanowicz

**Affiliations:** 1 Pediatric Surgery Department, Medical University of Bialystok, 15-089 Bialystok, Poland; ahermanowicz@wp.pl; 2 Independent Researcher, 15-483 Bialystok, Poland; izabela.cwalina20@gmail.com; 3 Biophysics Department, Medical University of Bialystok, 15-222 Bialystok, Poland; marzena.tylicka@umb.edu.pl; 4 Independent Researcher, 15-337 Bialystok, Poland; kwawrzyn99@gmail.com; 5 Social and Preventive Dentistry Department, Medical University of Bialystok, 15-267 Bialystok, Poland; magdalena.nowosielska@umb.edu.pl; 6 Faculty of Chemistry, Bioanalysis Laboratory, University of Bialystok, 15-245 Bialystok, Poland; ania@uwb.edu.pl (A.S.); lukas.old95@gmail.com (Ł.O.); ewka@uwb.edu.pl (E.G.)

**Keywords:** matrix metalloproteinase, MMP-1, MMP-2, fibronectin (FN), saliva, caries, endodontic treatment, surgical extraction, human

## Abstract

Objectives: Composition of saliva reflects the condition of the oral cavity. The aim of the study: Investigation of the concentrations of MMP-1 (Matrix metalloproteinase-1), MMP-2 (Matrix metalloproteinase-2) and fibronectin in the saliva of patients planned for endodontic treatment or surgical extraction. Material and methods: Seventy-five patients with caries and 14 healthy subjects were included in the study. Subjects were divided into group 1, in which 50 patients were planned for endodontic treatment, and group 2, in which 25 patients were planned for surgical extraction. For the measurements, we used a surface plasmon resonance imaging biosensor. Results: We found higher levels of MMP-1, MMP-2 and fibronectin in the saliva of patients planned for dental treatment than in healthy donors. We found lower concentrations of MMP-2 in subjects planned for surgical extraction, than in patients planned for endodontic treatment; however, there were no such differences in salivary concentrations of MMP-1 and fibronectin. There were no statistically significant differences in MMP-1 concentrations in the saliva before and after any type of dental treatment, but contrary to that, we found a statistically significant decrease in MMP-2 concentrations after endodontic treatment and after surgical extraction. We found a significant rise in the concentrations of fibronectin after surgical extraction but not after endodontic treatment. Conclusions: The concentrations of MMP-1 and MMP-2 in the saliva of our patients with caries were increased in comparison to healthy individuals, but after the treatment—so sanation of the oral cavity—we noted a decrease in matrix metalloproteinases (MMPs) levels. MMPs can be found in gingival crevicular fluid and saliva, carious dentin and plaque. According to our observations, the main source of MMPs in patients with caries is probably carious dentin. Increase in the salivary levels of fibronectin (FN) after surgical extraction may be connected with soft tissue injury caused by surgical extraction. Our results are another example of the fact that higher salivary concentrations of MMP-1, MMP-2 and FN can reflect the health status of the oral cavity in patients with caries.

## 1. Introduction

Saliva is the most important fluid in the oral cavity, where reactions occur between food, glands secretions, microorganisms and tissues. Composition of saliva reflects the condition of the oral cavity, so it can provide useful biomarkers [1]. Saliva consists of gingival crevicular fluid, comparable to serum, and salivary glands secretions consisting of water (99.5%), organic components (0.3%) and non-organic components (0.2%) [2].

Matrix metalloproteinases (MMPs) are enzymes that degrade basement membrane components and extracellular matrix. MMPs are divided into six different classes depending on their structure and action: collagenases (MMP-1, MMP-8, MMP-13 and MMP-18) and gelatinases (MMP-2 and MMP-9) [3].

MMP-2 and MMP-9 have a domain that binds gelatin and consists of three repeats of fibronectin type II. MMPs take part in physiological processes, including remodeling of tissues, healing of wounds, inflammation, immunity and angiogenesis [4,5]. MMPs are synthesized and secreted by connective tissue cells: fibroblasts, odontoblasts and osteoblasts [4,5]. Abnormal levels of MMPs indicate tissue destruction. Moreover, MMPs process bioactive substrates e.g., cytokines with anti-inflammatory action, growth factors, serum elements and chemokines, and in this way they affect anti-inflammatory and immune reactions [4,5,6]. The action of MMPs was discovered in various pathologic conditions such as atheroma, arthritis, cancer, periodontitis, tissue ulcers and fibrosis [4,5,6].

Saliva contains several MMPs, including collagenases and gelatinases derived from either the gingival crevicular fluid or the secretion of salivary glands. MMP-9 is the most abundant as it is derived from both sources [7]. Many studies have shown that salivary MMPs may have a strong contribution to dentin matrix degradation during the caries process. In our study, we concentrated on selected matrix metalloproteinases—MMP-1 and MMP-2. MMP-1 breaks down the extracellular matrix (ECM) by the cleavage of type I, II and III collagens, and MMP-2 (collagenase type IV) destroys collagen type IV, the main glycoprotein component of the basement membrane, and is involved in the regulation of vascular and inflammatory processes. Carious lesions were also found to contain both latent and active forms of MMP-3, MMP-2 and MMP-8 [8,9,10].

MMPs activity depends on the equilibrium between synthesis and their inhibiting factors: tissue inhibitors of metalloproteinases (TIMPs). During catalysis of MMPs, their proenzymes are activated and TIMPs are inhibited. In the oral cavity, MMPs can be found in gingival crevicular fluid (GCF) and saliva, carious dentin and plaque. MMPs are produced by macrophages present in GCF [5,11]. Gingival crevicular fluid is also the source of TIMPs, which are produced by B-cells [12]. GCF also contains α2-macroglobulin, a non-specific inhibitor of MMP, keeping a latent form of the MMPs in a balanced oral environment [4,13]. Expression of MMPs in relation to TIMPs reflects the degree of destruction of the tissue [5,14]. During caries decay, dissolving of mineral dentin exposes the organic matrix for destruction by bacteria and MMPs [4,5]. Bacteria produce acids that decrease pH, and in this way activate pro-MMPs [4,5]. Gelatinases and also collagenases were found in whole saliva. Periodontitis activates collagenases, which are normally present as a latent form [4,5]. MMP-1 and MMP-8 were found in saliva, regardless of the periodontal status of the patient [4,5].

## 2. Fibronectin

Fibronectin (FN) is a glycoprotein existing in two forms—soluble (in body fluids) and insoluble (in cells) [14]. A cellular isoform of FN is connected with the extracellular matrix and mediates cell–cell and cell–biomatrix attachments and is crucial for cell migration and organ differentiation [14,15]. FN is a large molecule so it cannot cross the acinar cells barrier [16]. According to Huynh et al. particles of FN found in GCF are the result of cleavage of FN by proteases such as MMPs during inflammation, wound healing and infections [17]. Fragments of FN may be used as an indicator of oral cavity status. Kapila et al. proved that FN and specific FN fragments induce the expression of proteinases in periodontal ligament cells, causing tissue degradation during periodontal disease [18]. A proapoptotic FN matrix induces ubiquitination and degradation of p53 in the proteasome, so FN molecules contribute to apoptosis associated with inflammation [19]. Higher levels of FN were detected in acute infections and also chronic diseases such as liver cirrhosis or hepatic carcinoma [20]. FN in saliva is a non-specific defense factor and is capable of binding to bacteria, thus contributing to bacterial plaque formation [21,22]. FN is thought to be responsible for bacterial aggregation [20].

FN also interacts with the glycoproteins of the human immunodeficiency virus (HIV) capsule and curbs its transmission [20,23]. FN can slow down cell death in fibroblast cultures, induced by hydrogen peroxide [20,23]. The concentrations of FN in saliva of patients with oral lichen planus are decreased in comparison to the general population [20]. FN isoform correlates with the existence of different types of tumors e.g., squamous cell carcinoma in the oral cavity [24].

Current interest in MMPs and fibronectin in saliva suggested the necessity for further investigations. Therefore, in our study we wanted to investigate the levels of MMP-1, MMP-2 and fibronectin in the saliva of patients with caries, planned for endodontic treatment or surgical extraction using the Surface Plasmon Resonance (SPR) imaging method [25,26,27,28].

## 3. Material and Methods

### 3.1. Patients

After screening, we included 75 patients with caries and 14 healthy volunteers in the study. The inclusion criteria were general good health and good oral hygiene. Exclusion criteria were smoking and any long-term medication. Informed consent in written form was collected from all participants. The study had the approval of the local ethics committee—Ethics Committee of Medical University of Bialystok, Poland No R-I-002/41/2019. Participants were split into two groups depending on the type of procedure planned for treatment. In group 1, 50 patients were planned for endodontic treatment with advanced caries, and in group 2, 25 patients were planned for surgical extraction. Group 1 (33 females and 17 males) had a mean age (SD) of 34.6 (2.6) years and group 2 (13 females and 12 males) had a mean age (SD) of 28.0 (2.9) years.

In group 1, patients needed endodontic treatment due to: simple caries, uncomplicated pulpitis classes I, II, III, IV, V according to Black’s Caries Classification.

In patients from group 2, indications for surgical extraction were acute and chronic acute inflammations: inflammation of the periapical tissues; submucosal and subperiosteal abscesses; tooth root cracks; complications after endodontic treatment; internal, perforating and external inflammatory resorptions; periodontal abscesses in the course of periodontal diseases; inflammation of the periapical tissues; pulp necrosis in the course of complicated caries.

The control group comprised 14 students (7 females and 7 males) with a mean age (SD) of 22.3 (1.6) years. Non-stimulated saliva was harvested before and after the intervention, and immediately centrifuged at 10,000× *g* for 5 min, and the supernatants were frozen and stored at −80 °C. Interviews, examinations of the oral cavity and collection of saliva were completed by the same doctor.

### 3.2. Methods

Saliva collection: Our patients were asked to abstain from consuming food and beverages, except water, for two hours before saliva collection. Fifteen minutes before the planned procedure, patients were seated in a chair and resting whole saliva samples were collected in plastic tubes and placed on ice for 15 min, under the control of a dentist, by the passive spitting method. The procedure was repeated 15 min after the endodontic or surgical procedure.

For the measurements of MMP-1, MMP-2 and FN levels we used a highly selective surface plasmon resonance imaging (SPRI) biosensor described elsewhere [26,27,28,29]. For MMP-1 measurements, the main part of the biosensor was an immobilized rabbit anti-human matrix metalloproteinase-1 antibody, binding the enzyme from the sample [27]. For MMP-2 measurements, Matrix metalloproteinase-2 specific inhibitor, ARP 101, was used as the receptor to bind the enzyme from the sample [26]. The biosensor for FN used the specific reaction of rabbit anti-fibronectin antibody [28]. To evaluate the results, the analyses of MMP-1, MMP-2 and FN levels in the biological samples were performed using enzyme-linked immunosorbent assay (ELISA), and we found good correlations between the results achieved using the SPRI biosensor and the commercial ELISA test (e.g., for MMP-2, correlation coefficients for healthy donors was 0.996, and for patients 0.984) [26,27,28]. 

### 3.3. Statistical Analysis

MMP-1, MMP-2 and FN activity is described as median with 25th and 75th percentiles. Because the MMP-1, MMP-2 and FN activity in the plasma of our patients did not pass the normality test, the Mann–Whitney U test and the Kruskal–Wallis H test were used to compare differences between groups. Statistical analyses were calculated with the STATISTICA PL release 10.0 program. A two-tailed *p* < 0.05 was considered significant.

## 4. Results

**Figure 1:**
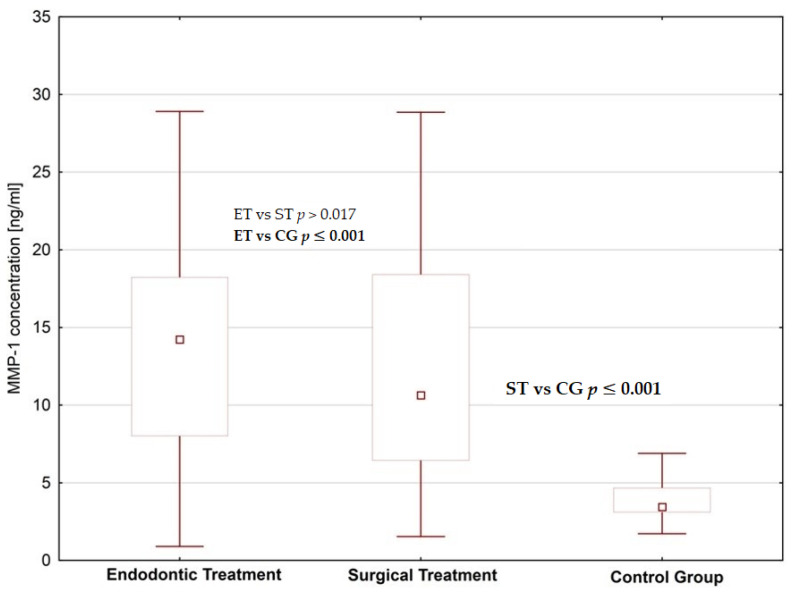
Saliva MMP-1 concentrations in patients planned for endodontic and surgical dental treatment in comparison to the control group.

**Figure 2:**
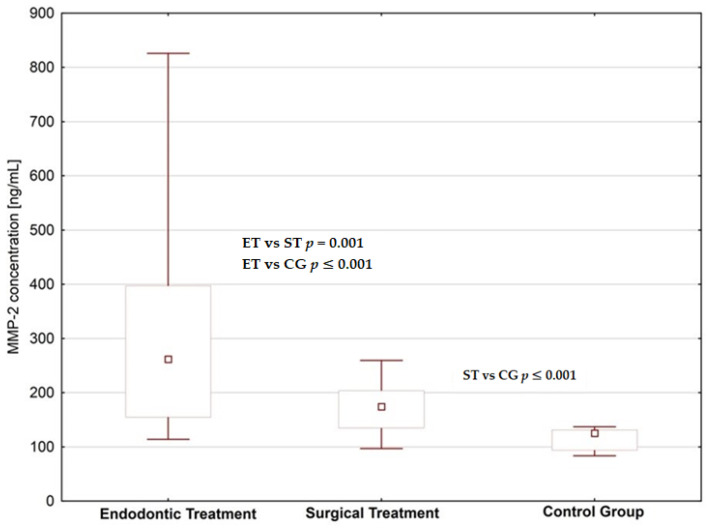
Saliva MMP-2 concentrations in patients planned for endodontic and surgical dental treatment in comparison to the control group.

**Figure 3:**
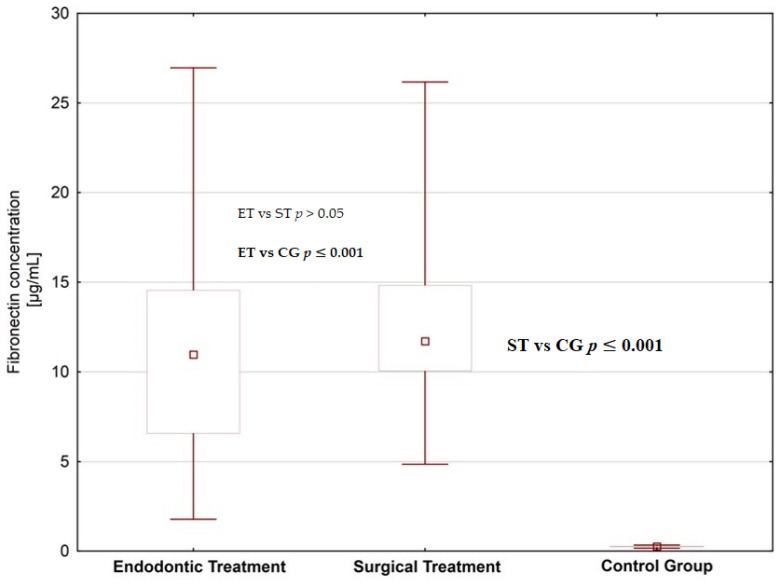
Saliva Fibronectin concentrations in patients planned for endodontic and surgical dental treatment in comparison to the control group.

We found increased concentrations of MMP-1, MMP-2 and fibronectin in the saliva of subjects planned for dental treatment (endodontic treatment and surgical extraction) than in healthy donors; the difference was statistically significant (Figure 1, Figure 2 and Figure 3, Table 1 and Table 2). When comparing patients planned for different dental procedures, we found lower concentrations of MMP-2 in patients planned for surgical extraction than in patients planned for endodontic treatment (*p* = 0.001); however, we did not find such differences in salivary levels of MMP-1 and fibronectin. 

**Figure 4:**
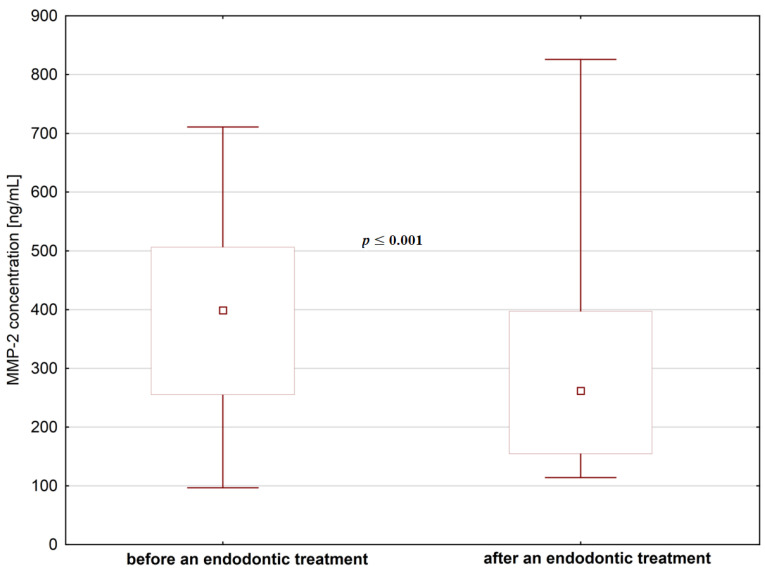
The concentrations of MMP-2 in saliva before and after endodontic treatment.

**Figure 5:**
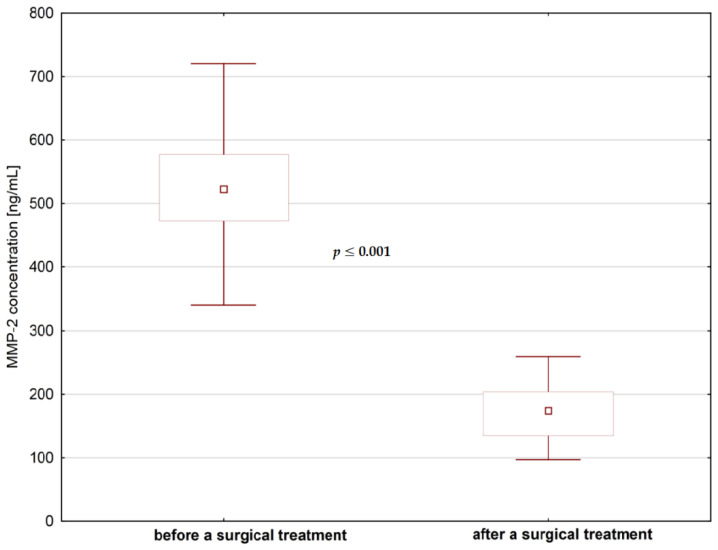
The concentrations of MMP-2 in saliva before and after surgical treatment.

**Figure 6:**
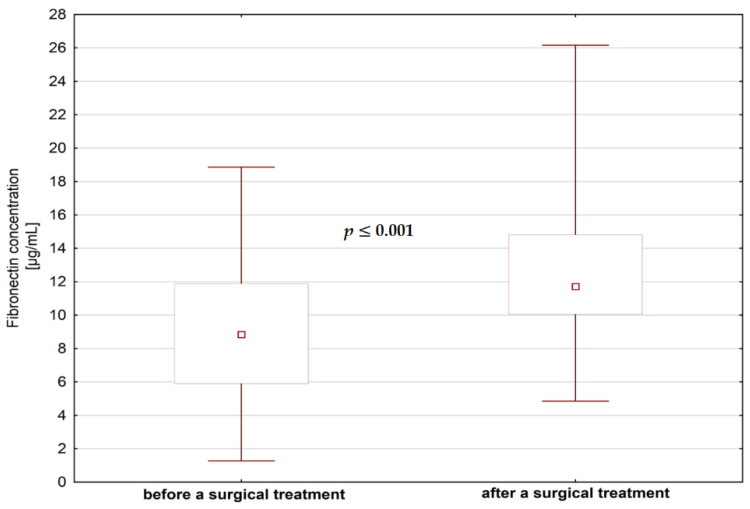
The concentrations of fibronectin in saliva before and after surgical treatment.

We did not find significant differences in MMP-1 concentrations in saliva before and after any type of dental treatment (*p* > 0.05), but contrary to that, we found a statistically significant decrease of MMP-2 levels after endodontic treatment and after surgical extraction (Figure 4 and Figure 5). As for the fibronectin, we found a statistically significant increase in the levels of fibronectin after surgical extraction but not after endodontic treatment (Figure 6).

Before the dental treatment, we did not find any correlation between MMP-1 and MMP-2 levels in saliva, but there was an average positive correlation between MMP-1 and MMP-2 concentrations in saliva after dental treatment.

We also found a negative weak correlation between MMP-2 and fibronectin concentrations after dental treatment.

## 5. Discussion

Testing of saliva is non-invasive, cheap and easy. Salivary composition can change quickly, so its analysis could be important in monitoring the severity of the diseases [1,2]. Samples of saliva should be taken after dental examination because bleeding from the gums can cause the contamination of samples [1,2].

MMPs in saliva reflect the degree of destruction of tissues depending on periodontal status and caries [5,30]. The same observation was done in our patients—we found increased levels of MMP-1 and MMP-2 in the saliva of individuals with caries, planned for dental treatment. Many studies have implied that MMPs take part in the devastation of dentin that follows demineralization caused by bacterial acids [4,5].

MMP production can be triggered by cytokines, growth factors, mechanical stress and changes in the extracellular matrix, leading to modification in cell–matrix interactions [31]. According to Overall et al. MMPs are activated by serine proteases (such as plasmin, tissue kallikrein and furin), by bacterial proteinases or by other members of the MMP family [4,31]. Nagase et al. found that pro-MMPs can also be activated by non-proteolytic agents, such as SH-reactive agents, mercurial compounds, acids, reactive oxygen and denaturants [32]. According to Jäsberg, MMP activity is regulated by the presence of endogenous MMP inhibitors—α2-macroglobulin and the TIMPs [4,5].

The essential function of MMPs is dissolving of the extracellular matrix (ECM), thus permitting remodeling of tissues. The ECM forms a structural barrier, but is also the source of biologically active molecules. Studies on animals have shown that, at the onset of dentinogenesis, MMP-2 expression is low and gradually increases during dentin formation [4]. Destruction of the ECM caused by MMPs changes its function and discharges active molecules. MMPs control the function of cytokines and chemokines, and influence homeostasis of tissues, host defense system and inflammation [4,33,34,35]. MMPs also activate certain growth factors [4,36,37].

MMP-1, MMP-2, stromelysin-1 (MMP-3) and MMP-9 were detected in dentin, odontoblasts and in the saliva, so it is estimated that those MMPs take part in the degradation caused by caries [4,38,39,40,41].

MMP-2 degrades collagen type IV, laminins and proteoglycans—elements forming basement membrane. Inactive precursors of MMPs in vitro are activated by MT 1–3 MMPs, plasmin, MMP-3 and by chemicals e.g., salts of mercury [6]. In dental caries, MMPs are activated by proteinases produced by bacteria [4]. Cathepsin B and L activated in saliva by mild acidity (pH 5–6.5) destroy collagen type I [4,7]. Cathepsins B and L also initiate degradation of matrix and activate MMPs [4]. Current research has found a correlation between MMPs and cathepsin B activity in saliva and has suggested that active cathepsin may result in an increased activation of MMPs [7]. High temperature and lower pH likewise activate MMPs [4].

In the saliva of patients with periodontitis, increased concentrations of MMP-2, MMP-8 and MMP-9 were found in comparison to healthy patients [7]. Contrary to that, according to Ingman et al., the concentrations of MMP-1 in saliva were comparable in patients and controls [42]. Interestingly, according to Nascimento et al., MMPs activity in the saliva is higher in patients with active, compared to chronic, carious lesions [43]. Contrary to that observation, we found lower concentrations of MMP-2 in patients planned for surgical extraction with active carious lesions than in patients planned for endodontic treatment with chronic caries. However we did not find such differences in salivary levels of MMP-1. Our observation is in line with the results found by Tersariol et al., who noticed a slight decrease in MMPs activity correlating with increasing depth of the lesion. Those facts support the speculation that dentin-bound, rather than pulp-derived MMPs are the major source for MMP activity in caries [44].

According to Nascimento et al., MMPs activity is age-dependent [42]. The aforementioned author found that MMPs activity decreased with age in both active and chronic lesions [42]. In our study, the mean age of patients was comparable in both studied groups, so it excludes potential age bias.

According to our observations, the concentrations of MMP-1 and MMP-2 in the saliva of patients with caries were increased in comparison to healthy individuals, but after the treatment—so sanation of the oral cavity—we noted a decrease in MMPs levels. In the case of MMP-2, the difference was statistically significant. This fact is further proof that salivary levels of MMPs can reflect the status of the oral cavity, although in the case of MMP-1 the difference between the concentrations before and after the dental treatment was statistically insignificant.

Furthermore, there are experimental data indicating that the diminishing of dentinal fluid flow may reduce dentinal caries progression [45,46], but because the concentrations of MMP-1 and MMP-2 in the saliva of our patients with caries were increased in comparison to healthy individuals, and after the treatment we noted a decrease in MMPs, we speculate that the main source of MMPs in patients with caries is probably carious dentin.

FN is a trace protein in saliva in physiologic conditions [24]. The levels of FN in saliva are higher when the permeability of the cellular barrier to plasma proteins is changed after tissue injury, or if the cellular FN from the extracellular matrix or ductal cells is released by hydrolytic cleavage [24]. Consequently, it was not surprising that levels of FN in saliva of patients planned for dental treatment—endodontic and surgical extraction, were higher than in healthy subjects. Nevertheless, in the literature there are contrary observations—according to Llena Puy et al. concentrations of FN in the saliva of patients with periodontal disease are decreased in comparison to healthy individuals [20]. According to some authors, the amount of FN fragments present in pathological saliva correlates with the acuteness of the disease [20]. In addition, it is known that FN is responsible for binding of Porphyromona gingivalis fimbriae [15]. The fact that we found a statistically significant increase in the salivary levels of FN after surgical extraction but not after endodontic treatment is probably caused by higher permeability of the cellular barrier caused by soft tissue injury after surgical procedure.

## 6. Conclusions

The concentrations of MMP-1 and MMP-2 in the saliva of our patients with caries were increased in comparison to healthy individuals, but after the treatment—so sanation of the oral cavity—we noted decrease in MMPs levels. MMPs can be found in gingival crevicular fluid and saliva, carious dentin and plaque. According to our observations, the main source of MMPs in patients with caries is probably carious dentin. Increase in the salivary levels of FN after surgical extraction may be connected with soft tissue injury caused by surgical extraction. Our results are another example of the fact that higher salivary concentrations of MMP-1, MMP-2 and FN can reflect the health status of the oral cavity in patients with caries.

## Figures and Tables

**Table 1:** The statistical parameters of MMP-1 and MMP-2 concentrations in saliva before and after dental treatment.

Value	MMP-1 Concentration (ng/mL)		MMP-2 Concentration (ng/mL)	
	Before Dental Treatment	After Dental Treatment	Before Dental Treatment	After Dental Treatment
Median	12.27	13.48	438.62	204.70
Minimum	2.45	0.90	96.72	97.04
Maximum	21.78	28.91	720.43	825.95
Percentiles (25–75%)	8.72–16.94	7.37–18.23	338.68–532.02	152.78–318.12
*p*-value *	*p* > 0.05		*p* ≤ 0.001	
Difference **	1.1		2.14	

* A *p* value < 0.05 is considered to show a significant difference between groups (according to Wilcoxon matched pairs test) ** (highest value/lower value).

**Table 2:** The statistical parameters of fibronectin concentrations in saliva before and after dental treatment.

Value	Fibronectin Concentration [µg/mL]	
	Before Dental Treatment	After Dental Treatment
Median	9.97	11.43
Minimum	1.27	1.77
Maximum	21.48	26.96
Percentiles (25–75%)	6.91–13.33	8.51–14.56
*p* value *	*p* = 0.057	
Difference **	1.15	

* A *p*-value < 0.05 is considered to show a significant difference between groups (according to Wilcoxon matched pairs test), ** (highest value/lower value).
